# Neuroimaging Biomarkers Predicting the Efficacy of Multimodal Rehabilitative Intervention in the Alzheimer’s Dementia Continuum Pathology

**DOI:** 10.3389/fnagi.2021.735508

**Published:** 2021-11-22

**Authors:** Sonia Di Tella, Monia Cabinio, Sara Isernia, Valeria Blasi, Federica Rossetto, Francesca Lea Saibene, Margherita Alberoni, Maria Caterina Silveri, Sandro Sorbi, Mario Clerici, Francesca Baglio

**Affiliations:** ^1^IRCCS Fondazione Don Carlo Gnocchi ONLUS, Milan, Italy; ^2^Department of Psychology, Università Cattolica del Sacro Cuore, Milan, Italy; ^3^Fondazione Policlinico Universitario “Agostino Gemelli” IRCCS, Rome, Italy; ^4^Università degli Studi di Firenze, NEUROFARBA, Firenze, Italy; ^5^Department of Physiopathology and Transplants, Università degli Studi di Milano, Milan, Italy

**Keywords:** neurodegenerative diseases, dementia, rehabilitation, biomarker, MRI, brain reserve, cognitive reserve

## Abstract

In this work we aimed to identify neural predictors of the efficacy of multimodal rehabilitative interventions in AD-continuum patients in the attempt to identify ideal candidates to improve the treatment outcome. Subjects in the AD continuum who participated in a multimodal rehabilitative treatment were included in the analysis [*n* = 82, 38 Males, mean age = 76 ± 5.30, mean education years = 9.09 ± 3.81, Mini Mental State Examination (MMSE) mean score = 23.31 ± 3.81]. All subjects underwent an MRI acquisition (1.5T) at baseline (T0) and a neuropsychological evaluation before (T0) and after intervention (T1). All subjects underwent an intensive multimodal cognitive rehabilitation (8–10 weeks). The MMSE and Neuropsychiatric Inventory (NPI) scores were considered as the main cognitive and behavioral outcome measures, and Delta change scores (T1–T0) were categorized in Improved (ΔMMSE > 0; ΔNPI < 0) and Not Improved (ΔMMSE ≤ 0; ΔNPI ≥ 0). Logistic Regression (LR) and Random Forest classification models were performed including neural markers (Medial Temporal Brain; Posterior Brain (PB); Frontal Brain (FB), Subcortical Brain indexes), neuropsychological (MMSE, NPI, verbal fluencies), and demographical variables (sex, age, education) at baseline. More than 50% of patients showed a positive effect of the treatment (ΔMMSE > 0: 51%, ΔNPI < 0: 52%). LR model on ΔMMSE (Improved vs. Not Improved) indicate a predictive role for MMSE score (*p* = 0.003) and PB index (*p* = 0.005), especially the right PB (*p* = 0.002) at baseline. The Random Forest analysis correctly classified 77% of cognitively improved and not improved AD patients. Concerning the NPI, LR model on ΔNPI (Improved vs. Not Improved) showed a predictive role of sex (*p* = 0.002), NPI (*p* = 0.005), PB index (*p* = 0.006), and FB index (*p* = 0.039) at baseline. The Random Forest reported a classification accuracy of 86%. Our data indicate that cognitive and behavioral status alone are not sufficient to identify best responders to a multidomain rehabilitation treatment. Increased neural reserve, especially in the parietal areas, is also relevant for the compensatory mechanisms activated by rehabilitative treatment. These data are relevant to support clinical decision by identifying target patients with high probability of success after rehabilitative programs on cognitive and behavioral functioning.

## Introduction

Cognitive disability affects 10.8% of adults living with a chronic condition, and is characterized by a complex impairment in attention, memory and/or decision making. With the aging of the general population (World Health Organization, 2012) cognitive disabilities in the adult are often observed as clinical signs of neurodegenerative diseases as in Alzheimer’s continuum conditions, ranging from Mild Cognitive Impairment (MCI) to Alzheimer’s Dementia (AD) (Aisen et al., 2017; Jack et al., 2018).

MCI is a mild neurocognitive disorder (American Psychiatric Association, 2013; Stokin et al., 2015), affecting 6–25% of people aged over 60, characterized by isolated impairment in one or more cognitive processes, often involving memory (amnestic MCI), with a complete autonomy in functional activities of daily living (Langa and Levine, 2014; Petersen et al., 2018). Each year, 5–25% of amnestic MCI individuals develop AD (Hänninen et al., 2002; Grundman et al., 2004), thus experiencing a worsening of cognitive abilities, gradual loss of functional autonomies and different degrees of behavioral and psychological symptoms (Lyketsos et al., 2000, 2002) such as depression, agitation, apathy and delusions (Cummings, 2004; Steinberg et al., 2008). Especially, behavioral symptoms associated to AD impact seriously on patient’s management in daily living, as well as caregiver distress (Steinberg et al., 2008). Unfortunately, behavioral changes represent a mark of the disease and is strictly linked with the need of hospitalization (Spector et al., 2013; Maki et al., 2018).

Clinical, neuropsychological and behavioral aspects of AD continuum are, in its typical form, paralleled by the pathophysiological counterpart of the disease: a progressive accumulation and spreading of amyloid plaques and neurofibrillary T-tau protein tangles starting even years before the clinical onset of symptoms (Aisen et al., 2017; Ekman et al., 2018). The pathology starts in the medial temporal lobe and limbic areas (enthorinal cortex, hippocampus, parahippocampal regions) and reaches associative cortices (Braak and Braak, 1991). Different patterns of cortical atrophy are associated with the diffusion of tangles in the brain, such as the earlier involvement of posterior-parietal regions (Lehmann et al., 2013; Ekman et al., 2018) or the presence of frontal lobe atrophy in “executive AD” presentation (Ferreira et al., 2016). Given this progression, specific brain changes such as hippocampal atrophy rates and local atrophy indices are established neuroimaging biomarkers of AD-associated downstream neuronal degeneration (McKhann et al., 2011; Ekman et al., 2018). To date, AD is the most diffuse form of dementia, affecting globally 4.7 million individuals aged 65 + and a projected rise to 130 million individuals worldwide by 2,050 (Lopez and Kuller, 2019).

Despite the great efforts spent in clinical and translational research, the possible effect of symptomatic drugs on patients suffering from AD continuum remains controversial (Birks, 2006; Birks and Grimley Evans, 2015) with a single molecule, Aducanumab, recently obtaining the FDA approval (Fillit and Green, 2021) and new disease-modifying pharmacological treatments still in clinical developing stages (Sabbagh et al., 2020).

The most-adopted intervention is thus rehabilitation, tested in manifold settings: from single-cognitive-domain approaches to the most recent holistic multi-modal interventions (Fabbri et al., 2018; Maki et al., 2018), amply documented to be effective in neurodegenerative conditions (Baglio et al., 2015; Chew et al., 2015; Realdon et al., 2016; Isernia et al., 2019). In fact, considering the difficulties faced by individuals in the AD continuum, often impacting cognitive and behavioral functionality, multidisciplinary models of care are taken in consideration to manage such a great variety of symptoms. Multidisciplinary approaches have the advantage to mutually complement and optimize benefits on different target of rehabilitation (Maki et al., 2018). Importantly, the main effects of multimodal approaches are demonstrated in several domains, including daily living skills, global physical functions and cognition (McDermott et al., 2019). These effects are the results of multi-domain cognitive stimulation, motor enhancement and occupational activities which are implemented in the framework of a multidisciplinary clinical team. This complex setting drives a parallel action on both enhancement and maintenance of cognitive residual abilities, attaining and aligning with the values of the International Classification of Functioning, Disability and Health and supporting the quality of life of people regardless their level of functioning (Gitlin et al., 2013; World Health Organization, 2017; Maki et al., 2018).

To our knowledge, to date no clear evidences have been proposed to forecast which patients can mostly benefit from these rehabilitation treatments. This can be partially explained by the observation that, despite the known neuropathological progression of the disease, a disjunction between brain damage and clinical outcome is often observed, accounting for individual differences in coping with the pathology (Williams et al., 2018). In particular, genetic, epigenetic and environmental factors can mitigate the effects of neural decline caused by aging and age-related diseases (Cabeza et al., 2018). Identifying which neuro-clinical features are prognostic of treatment success is urgent with potentially vast implications for the personalization of interventions and maximizing the effectiveness of rehabilitation programs. This would allow to *a priori* differentiate between people who potentially benefit from the treatment and those who not.

Concepts such as cognitive and brain reserves (Stern, 2009) can catch the individual differences both in how people process cognitive tasks and in how their brains can morphologically differ each other, aspects well known to be mediated by life experiences (Maguire et al., 2006; Stern, 2009). Cognitive reserve has been defined as the processing resources gained over time as a result of engaging in mentally stimulating activities, i.e., education, professional attainment, and leisure activities (Stern, 2009). Although the relevance to measure cognitive reserve, a plethora of “convenience proxies” to operationalized this construct, as socio-behavioral indices, such as education, has been reported (Stern, 2009). In this framework, brain reserve is hypothesized to be the result of the accumulation of neural resources before the brain is affected by age-related processes, over a period of years (Cabeza et al., 2018). Brains with higher reserve can sustain more insult before clinical deficit emerges, and thus individual differences in brain reserve can led to differences in the clinical expression of a particular degree of damage to the brain (Stern, 2009). Brain reserve has been operatively quantified in terms of functional or morphometric measures (gross whole-brain measures reflective of peak or premorbid brain volume, including Total Intracranial Volume or head circumference) (Katzman et al., 1988; Stern, 2009). Ongoing research has begun to incorporate more finegrained measures such as specific patterns of gray matter volume, cortical surface area, and cortical thickness. Changes after treatment have been reported, such as changes in medial temporal lobe structures in subjects that performed intensive mnestic training (Maguire et al., 2006), but the detection of specific neural structures as critical hub of neural reserve has not yet been demonstrated.

Despite the association between a good brain reserve and the increased probability to positively cope with neural injuries, to date no clear indications can forecast the effects of a given brain reserve on the results of a rehabilitative intervention, and the prognostic characteristics of treatment success still remain a matter of debate. A better knowledge of the prognostic neural profile of rehabilitation candidates, in terms of level of probability of treatment effectiveness, could be beneficial both for individual patients, who would receive a more efficacious intervention, and for the healthcare system.

In this work we aim to identify the best candidates for effective rehabilitative interventions in AD-continuum disease patients. We included neuroimaging biomarkers as aspects of brain reserve and, in line with our previous work (Di Tella et al., 2020), we used classification approaches including Random Forest and logistic regression to define which neural (brain reserve), demographical and clinical aspects of the disease might predict the best outcome for multimodal rehabilitation. Given the literature supporting the role of brain reserve on clinical expression of diseases and deficits (Stern, 2009), we hypothesize to find a significant predictor of neurorehabilitation success in critical hub of neural reserve, such as specific morphometric volumes.

## Materials and Methods

All the patients with a diagnosis in the AD-continuum, consecutively admitted to the Memory Clinic of IRCCS Fondazione Don Carlo Gnocchi ONLUS, Centro Santa Maria Nascente (Milan) from 2011 to 2019 and fulfilling the admission criteria (see below) had the possibility to participate in a multimodal rehabilitation treatment. This IRCCS Don Carlo Gnocchi is a scientific institute for rehabilitation and research with a specific focus on neurodegenerative diseases. For this reason, all subjects at admission are asked to provide an informed consent (by signing the informed consent module approved by Don Gnocchi Foundation Ethics Committee) allowing the use of clinical data collected during evaluation and rehabilitation for research purposes. No procedures different from standard were performed for the present study.

Admission criteria were: (1) a diagnosis of an AD-continuum condition, from MCI to mild-to- moderate AD according to National Institute on Aging-Alzheimer’s Association guidelines (Albert et al., 2011; McKhann et al., 2011) reported in the clinical documentation; (2) age ≥ 65 years old; (3) minimum education level being alphabetization (2 years); (4) right-hand dominance (Oldfield, 1971); (5) attendance of a multimodal intensive rehabilitation intervention tailored for mild-to-moderate stages of AD continuum followed at IRCCS Santa Maria Nascente for at least 80% of program’s sessions (see below); (6) presence of a MRI examination not earlier than 2 months before the beginning of rehabilitation treatment; (7) presence of a neuropsychological evaluation pre- and post- intervention; (8) a stable pharmacological treatment (acetylcholine esterase inhibitors and neuropsychiatric drugs, if any) at least for 3 months before starting the rehabilitation. Exclusion criteria were indeed considered: (1) presence of a prodromic condition or a diagnosis of other types of dementia different from AD-continuum; (2) presence of major psychiatric disorders; (3) absence of a written informed consent.

All patients fulfilling the criteria were admitted in an intensive rehabilitation program (8–10 weeks, 3–5 times a week) based on a holistic approach (Baglio et al., 2015; Fabbri et al., 2018). Rehabilitation was conceived to train cognition by enhancing several domains (cognition, physical, and social) *via* neuropsychological activities (both paper-and-pencil and computerized tasks addressing different cognitive domains, such as memory, executive functions, language, attention, abstraction, praxis), psychomotor exercises (stretching, postural changes, gait exercises, balance, and postural control), and recreational/occupational activities (functional and goal-based exercises in order to readapting the use of daily tools and performing everyday tasks to recover personal autonomy and to improve targeted domains of quality of life) were proposed. By training different domains of functioning, the treatments aimed to act in an integrated manner on residual cognitive functions of AD-continuum people, triggering neuroplasticity mechanisms (e.g., Venna et al., 2014). The principal setting of the intervention was in a small group (2–4 person) with a therapist who helped the rehabilitation program for patients, and the dose of the treatment was intense: about 3–5 times a week, about 60-min per session. The programs were based on multi-stimulation therapy (Baglio et al., 2015) and a multidisciplinary rehabilitation team (physiotherapist, neuropsychologist, and occupational therapist) cooperated in the rehabilitation plan implementation and monitoring.

Retrospectively, demographic and clinical data have been extracted from clinical charts by a single researcher (FR) and inserted in an anonymized database, subsequently used for the statistical analyses of the present research. The database included, for each recruited subject:

- Age, gender, education diagnosis and anamnesis (disease history and mood evaluation Hamilton, 1960);
- *Mini-Mental State Examination* (MMSE; Folstein et al., 1983) as index of the global cognitive level of patients. The total score, ranged 0–30, suggests the absence of cognitive impairment (MMSE score: 27–30), the presence of borderline impairment (MMSE score: 24–26), mild cognitive impairment (MMSE score: 18–23), moderate cognitive impairment (MMSE score: 14–17), or severe cognitive impairment (MMSE score: 0–13).
- *Verbal Fluencies* (Novelli et al., 1986; Carlesimo et al., 1996) assessing language and executive functions. In details, both letter (FAS) and categorical (CAT) fluencies were extracted from charts and included in the analysis. The raw total score of the test performance was adjusted for age and education following the instructions’ procedure of Novelli et al. (1986) and Carlesimo et al. (1996).
- *Neuropsychiatric Inventory* (NPI; Cummings et al., 1994; Cummings, 1997) as a measure of the frequency and severity of behavioral symptoms related to the clinical condition, including delusions, hallucinations, dysphoria, anxiety, euphoria, aggression, apathy, irritability, disinhibition, troublesome behavior. Both the scores of frequencies and severity of symptoms (NPI_f*s_) and distress of caregiver (NPI_distress_) were reported.

Moreover, from the MRI examinations (1.5T Siemens Magnetom Avanto scanner, Erlangen, Germany) acquired before the rehabilitation treatment, we retrieved anonymized conventional sequences to exclude gross brain abnormalities and a high-resolution T1-3D MPR (TR/TE = 1,900/3.37 ms; FoV = 192 × 256 mm, isometric in-plane resolution 1 mm, 176 axial slices) to assess brain morphometry.

### Statistics

#### MRI Data Analysis and Computation of Neuroimaging Biomarkers

To extract morphometrical data, MPR acquisitions have been analyzed using the recon-all pipeline of Freesurfer software (v.5.3).^1^ Quality check have been performed for each subject according to ENIGMA guidelines^2^ and manual corrections performed to improve automatic segmentation when necessary. Brain parcellation were performed according to Fischl et al. (2002) and Desikan et al. (2006) atlases. As neuroimaging biomarkers, volumetric measurements were computed considering brain areas strongly related to AD-continuum conditions, according to Ekman et al. (2018). In particular, we computed (a) Medial Temporal Brain (MTB) index (sum of volumes in: hippocampal and parahippocampal volumes); (b) Posterior Brain (PB) index (sum of volumes in: posterior cingulate, precuneus, superior parietal, inferior parietal, supramarginal gyrus); (c) Frontal Brain (FB) index (sum of volumes in: caudal middle frontal, rostral middle frontal, pars opercularis, pars triangularis, pars orbitalis, frontal pole, superior frontal, rostral anterior cingulate, caudal anterior cingulate, precentral, lateral orbito-frontal, medial orbitofrontal). In addition, we also computed a (d) Subcortical Brain (SBCB) index (sum of volumes in: thalamus, amygdala, nucleus accumbens, caudate nucleus) (Roh et al., 2011). In each subject, the brain neuroimaging biomarkers have been computed separately for left and right hemispheres, as well as globally (MTB_rh_, MTB_lh_, MTB_global_, PB_rh_, PB_lh_, PB_global_, FB_rh_, FB_lh_, FB_global_, SBCB_rh_, SBCB_lh_, SBCB_global_). All indices have been normalized to the estimated Total Intracranial Volume, and converted in *z*-values considering MRI mean and SD data from an age- gender- and education-matched sample of healthy controls (*n* = 32, 13 M, mean age 74.16 ± 4.33, internal laboratory dataset). These *Z*-values have been included in subsequent statistical analyses (Z-MTB_rh_, Z-MTB_lh_, Z-MTB_global_, Z-PB_rh_, Z-PB_lh_, Z-PB_global_, Z-FB_rh_, Z-FB_lh_, Z-FB_global_, Z-SBCB_rh_, Z- SBCB_lh_, Z- SBCB_global_).

#### Demographic, Clinical, and Behavioral Measures

Statistical analyses were performed with IBM SPSS Statistics software (version 24) and JASP (JASP Team 2020, JASP version 0.14.1). Means, frequencies, and standard deviations were computed to describe sample characteristics. χ^2^-test was used to verify if sex distribution and education were balanced in the whole sample.

The MMSE score was considered the primary clinical outcome measure for the cognitive status. Delta change score (T1–T0) of MMSE was categorized in Improved (ΔMMSE > 0) and Not Improved (ΔMMSE ≤ 0). The NPI_f*s_ was considered the primary clinical outcome measure for the behavioral status. Delta scores (T1–T0) of NPI was categorized in Improved (ΔNPI_f*s_ < 0) and Not Improved (ΔNPI_f*s_ ≥ 0).

Logistic Regression classification models including demographical characteristics (age, sex, years of education), neural markers (Z-MTB_global_, Z-PB_global_, Z-FB_global_, Z-SBCB_global_) and neuropsychological variables (MMSE T0, NPI_f*s_ T0, FAS T0, CAT T0) at baseline were performed to identify the subjects that significantly benefited from treatment (ΔMMSE > 0 and ΔNPI_f*s_ < 0) as in Di Tella et al. (2020). Wald forward option was used as a stepwise selection method (entry criterion *p* < 0.05, removal criterion *p* > 0.10). Only for cognitive outcome neural markers were split in left and right side.

For confirmatory purposes, Random Forest classification models were run including only predictors retained in the last step of the logistic regressions. We built Random Forest with the default parameter values in JASP (version 0.14.1), specifically with respect to data split we partitioned the data set into a training (60%), validation (20%), and test set (20%). In relation to the number of trees, we selected an optimal number of trees [Ntrees (maximum) = 100], optimized with respect to the out-of-bag accuracy. Performance of the classification model was evaluated by calculating the classification accuracy that represents the proportion of the instances that were classified correctly, summing up true positive and true negative cases.

## Results

### Demographical Characteristics of the Sample

In total, 82 people (38 males) with a diagnosis of AD-continuum condition (n_MCI_ = 54, n_AD_ = 28) were included in the study. Table 1 shows data referred to neuropsychological assessment and morphometrical z-scores of the computed brain neuroimaging biomarkers.

**TABLE 1:** Neuropsychological assessment and morphometrical z-scores of the sample.

	25th percentile	75th percentile	Mean	*SD*
Age (years)	72.25	79.00	76.00	5.30
Education (years)	6.00	13.00	9.09	3.81
Hamilton T0*	3.75	8.00	6.62	4.55
MMSE T0 (0–30)	21.00	26.00	23.32	4.08
FAS T0	16.25	30.50	23.10	10.55
CAT T0	18.00	30.75	24.33	9.96
NPI_f*s_ T0 (0–144)	4.75	19.50	12.75	9.47
NPI_distress_ T0 (0–60)	3.00	10.00	6.67	4.83
Z-MTB_global_	–2.70	–1.18	–1.86	1.43
Z-MTB_lh_	–2.72	–1.13	–1.88	1.39
Z-MTB_rh_	–2.73	–0.82	–1.75	1.54
Z-PB_global_	–2.54	–0.71	–1.54	1.38
Z-PB_lh_	–2.28	–0.49	–1.35	1.29
Z-PB_rh_	–2.54	–0.61	–1.58	1.43
Z-FB_global_	–2.82	–0.45	–1.56	1.80
Z-FB_lh_	–3.08	–0.50	–1.69	1.87
Z-FB_rh_	–2.52	–0.29	–1.40	1.72
Z-SBCB_global_	–1.21	–0.13	–0.65	0.84
Z-SBCB_lh_	–1.23	–0.15	–0.70	0.87
Z-SBCB_rh_	–1.16	–0.08	–0.58	0.87

Hamilton T0, Hamilton Depression Scale; MMSE T0, Mini-Mental State Examination at baseline; FAS T0, Phonological Fluency at baseline; CAT T0, Categorial Fluency at baseline; NPI_f*s_ T0, Neuropsychiatric Inventory frequencies and severity of symptoms at baseline; NPI_distress_ T0, Neuropsychiatric Inventory caregiver distress; Z-MTB_global_, Z-values of Medial Temporal Brain index; Z-MTB_lh_, Z-values of left Medial Temporal Brain index; Z-MTB_rh_, Z-values of right Medial Temporal Brain index; Z-PB_global_, Z-values of Posterior Brain index; Z-PB_lh_, Z-values of left Posterior Brain index; Z-PB_rh_, Z-values of right Posterior Brain index; Z-FB_global_, Z-values of Frontal Brain index; Z-FB_lh_, Z-values of left Frontal Brain index; Z-FB_rh_, Z-values of right Frontal Brain index; Z-SBCB_global_, Z-values of Subcortical Brain index; Z-SBCB_lh_, Z-values of left Subcortical Brain index; Z-SBCB_rh_, Z-values of right Subcortical Brain index.

*This data was available only for 64 participants.

### Response to the Treatment

The percentage of not responders to the treatment in both cognitive and behavioral outcome was 22% (see Table 2). Fifty-one percent of patients showed an improvement on global cognitive functioning after the treatment (ΔMMSE > 0: 51%; AD: 38%; MCI: 13%) showing a mean ΔMMSE = 2.24 (ΔMMSE_AD_ = 2.13; ΔMMSE_MCI_ = 2.55), Cohen’s *d* = 2.15. A reduction of behavioral symptoms after the treatment was observed in a large number of cases (ΔNPI_f*s_ < 0= 52%, AD: 32%; MCI: 20%), showing a mean ΔNPI_f*s_ = −5.60 (ΔNPI_f*sAD_ = −6.67; ΔNPI_f*sMCI_ = −3.81), Cohen’s *d* = 2.18 (Table 2). Baseline characteristics comparison between responders and not responders to rehabilitation program are reported in Supplementary Tables 1,2.

**TABLE 2:** Percentages of responders and not responders to the treatment in cognitive and behavioral outcomes.

	Not responders at NPI_f*s_ (%)	Responders at NPI_f*s_ (%)
Not responders at MMSE (%)	22.0%	26.8%
Responders at MMSE (%)	25.6%	25.6%

MMSE, Mini-Mental State Examination; NPI_f*s_, Neuropsychiatric Inventory frequencies and severity of symptoms at baseline.

### Improvement in the Cognitive Status

Significant logistic regression model (Wald method, Nagelkerke *R*^2^ = 0.229) on ΔMMSE (Improved vs. Not Improved) showed in the final second step a predicted role of MMSE score at baseline (*p* = 0.003) and Z-PB_global_ index (*p* = 0.005) (Table 3). Age, sex, educational years, FAS at baseline, CAT at baseline, NPI_f*s_ at baseline, Z-MTB_global_ index, Z-FB_global_ index, Z-SBCB_global_ index were excluded from the equation (*p* > 0.05).

**TABLE 3:** Binary logistic regression model to test best predictors of the MMSE change after rehabilitation.

		β	S.E.	Wald	*p*-value	Exp(β)	95% C.I. for Exp(β)	
							Lower	Upper
Step 1	MMSE baseline	–0.142	0.061	5.474	**0.019**	0.868	0.770	0.977
	Constant	3.358	1.439	5.444	0.020	28.727		
Step 2	MMSE baseline	–0.214	0.072	8.947	**0.003**	0.807	0.702	0.929
	Z-PB_global_ index	0.594	0.211	7.929	**0.005**	1.811	1.198	2.739
	Constant	6.006	1.870	10.310	0.001	405.674		

*MMSE T0, Mini-Mental State Examination at baseline; Z-PB_global_, Z-values of Posterior Brain index; S.E., standard error; C.I., confidence intervals. Relevant statistically significant results are reported in bold.*

When considering the left and right hemisphere separately, only MMSE score at baseline (*p* = 0.002) and Z-PB_rh_ index (*p* = 0.002) remained in the final second regression model (Nagelkerke *R*^2^ = 0.259) (Table 4). Age, sex, educational years, FAS at baseline, CAT at baseline, NPI_f*s_ at baseline, *Z*-values of Z-MTB_lh_ and Z-MTB_rh_ index, Z-PB_lh_ index; Z-FB_lh_ and Z-FB_rh_ index, Z-SBCB_lh_ and Z-SBCB_rh_ index were excluded from the equation (*p* > 0.05).

**TABLE 4:** Binary logistic regression model to test best predictors of the MMSE change after rehabilitation.

		β	S.E.	Wald	*p-value*	Exp(β)	95% C.I. for Exp(β)	
							Lower	Upper
Step 1	MMSE baseline	–0.142	0.061	5.474	**0.019**	0.868	0.770	0.977
	Constant	3.358	1.439	5.444	0.020	28.727		
Step 2	MMSE baseline	–0.229	0.074	9.633	**0.002**	0.795	0.688	0.919
	Z-PB_rh_ index	0.657	0.214	9.409	**0.002**	1.929	1.268	2.935
	Constant	6.478	1.940	11.151	0.001	650.428		

*MMSE, Mini-Mental State Examination; Z-PB_rh_, Z-values of right Posterior Brain index; S.E., standard error; C.I., confidence intervals. Relevant statistically significant results are reported in bold.*

**FIGURE 1:**
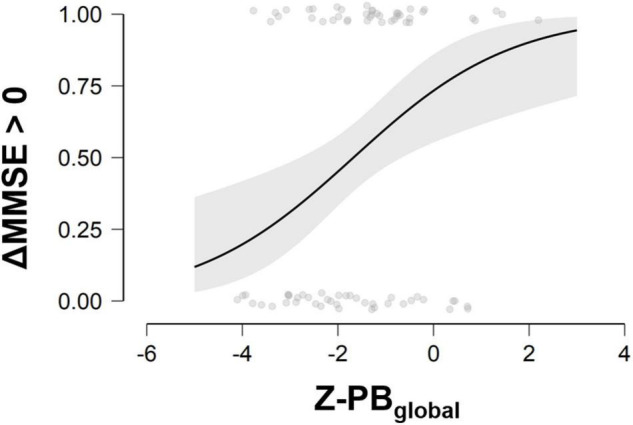
Probability to improve in the cognitive status at different scores of MMSE at baseline and Z-PB_global_. MMSE, Mini-Mental State Examination; ΔMMSE > 0, Delta change score (T1–T0) of Mini-Mental State Examination; Z-PB_global_, *Z*-values of Posterior Brain index. Three ranks of PB index values can be considered for interpretation purposes: Low PB: Z-score ranges −4.10 to −2.22; Intermediate PB: Z-score ranges −2.15 to −0.95; High PB: Z-score ranges −0.88 to 2.19.

When considering predicted probability of success, ideal candidate for the multimodal treatment was a person with lower MMSE at baseline and higher brain volume in PB-index, especially in the right PB-index (see Figure 1).

The Random Forest analysis, run to confirm the classification model, gave an accuracy score of approximately 77% including only variables retained at the last step of the regression model for the identification of participants who significantly benefited from the treatment and those which did not (Table 5).

**TABLE 5:** Random Forest results.

			Predicted	
	ΔMMSE		Not responders	Responders
Actual	Not responder	Count	28	12
		%	70.0%	30.0%
	Responder	Count	7	35
		%	16.7%	83.3%

*Confusion matrix summarizing the performance of the RF classification algorithm on the cognitive outcome. The column targets (Predicted Not responders/Responders) are predicted values by the RF and the row targets (Actual Not responders/Responders) are the actual values. Classification accuracy represents the proportion of the instances that were classified correctly (Actual Not responders and Predicted Not responders + Actual Responders and Predicted Responders/Total sample). MMSE, Mini-Mental State Examination.*

### Improvement in Behavioral Symptoms

Significant logistic regression model (Wald method, Nagelkerke *R*^2^ = 0.352) on ΔNPI_f*s_ (Improved vs. Not Improved) showed in the final fourth step a predicted role of sex (*p* = 0.002), ΔNPI_f*s_ at baseline (*p* = 0.005), Z-PB_global_ index (*p* = 0.006), and Z-FB_global_ (*p* = 0.039) (Table 6). Age, educational years, FAS at baseline, CAT at baseline, MMSE at baseline, Z-MTB_global_ index, Z-SBCB_global_ index were excluded from the equation (*p* > 0.05).

**TABLE 6:** Binary logistic regression model to test best predictors of the NPI f*s change after rehabilitation.

							95% C.I. for Exp(β)	
		β	S.0E.	Wald	*p*-value	Exp(β)	Lower	Upper
Step 1	Sex	–1.333	0.475	7.888	**0.005**	0.264	0.104	0.669
	Constant	0.762	0.324	5.545	0.019	2.143		
Step 2	Sex	–1.314	0.497	6.984	**0.008**	0.269	0.101	0.712
	NPI_f*s_ T0	0.070	0.029	5.879	**0.015**	1.073	1.014	1.136
	Constant	–0.117	0.470	0.062	0.803	0.889		
Step 3	Sex	–1.424	0.519	7.521	**0.006**	0.241	0.087	0.666
	NPI_f*s_ T0	0.079	0.030	7.051	**0.008**	1.082	1.021	1.147
	Z-PB_global_ index	0.428	0.204	4.388	**0.036**	1.534	1.028	2.288
	Constant	0.555	0.576	0.929	0.335	1.742		
Step 4	Sex	–1.758	0.580	9.175	**0.002**	0.172	0.055	0.538
	NPI_f*s_ T0	0.086	0.031	7.711	**0.005**	1.090	1.026	1.157
	Z-PB_global_ index	1.123	0.408	7.588	**0.006**	3.073	1.382	6.830
	Z-FB_global_ index	–0.605	0.294	4.243	**0.039**	0.546	0.307	0.971
	Constant	0.754	0.613	1.511	0.219	2.125		

NPI_f*s_, Neuropsychiatric Inventory frequencies and severity of symptoms at baseline; Z-PB_global_, Z- values of Posterior Brain index; Z-FB_global_, Z-values of Frontal Brain index; S.E., standard error; C.I., confidence intervals. Relevant statistically significant results are reported in bold.

**FIGURE 2:**
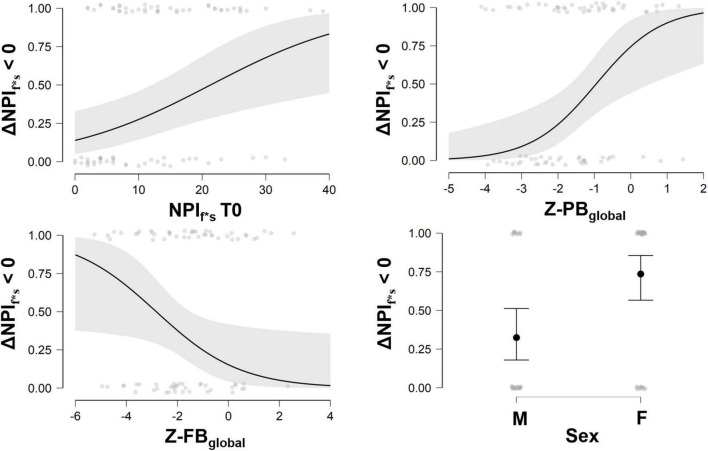
Probability to reduce behavioral symptoms at different scores of NPI_f*s_ at baseline, and Z-PB_global_, Z-FB_global_, and sex. NPI, Neuropsychiatric Inventory; ΔNPI_f*s_ > 0, Delta change score (T1–T0) of Neuropsychiatric Inventory frequencies and severity of symptoms; Z-PB_global_, *Z*-values of Posterior Brain index; Z-FB_global_, *Z*-values of Frontal Brain index; M, males, F, females. Three ranks values can be considered for interpretation purposes for PB index (Low PB: Z-score ranges −4.10 to −2.22; Intermediate PB: Z-score ranges −2.15 to −0.95; High PB: Z-score ranges −0.88 to 2.19) and FB index (Low FB: Z-score ranges −5.25 to −2.32; Intermediate FB: Z-score ranges −2.30 to −0.87; High FB: Z-score ranges −0.81 to 2.56).

When exploring predicted probability of success, ideal candidate for the multimodal treatment was a person with higher severity of NPI_f*s_ at baseline, lower brain volume in FB-index and higher brain volume in PB-index (see Figure 2).

Finally, for confirmatory purposes we ran Random Forest analysis. This analysis reported an accuracy of prediction approximately of 86% including demographical, neurostructural, and neuropsychological variables at baseline retained in the last step of the regression model for the identification of participants who significantly benefited from the treatment and those which did not (Table 7).

**TABLE 7:** Random Forest results.

			Predicted	
	ΔNPI_f*s_		Not responders	Responders
Actual	Not responders	Count	35	2
		%	94.6%	5.4%
	Responders	Count	9	34
		%	20.9%	79.1%

Confusion matrix summarizing the performance of the RF classification algorithm on the behavioral outcome. The column targets (Predicted Not responders/Responders) are predicted values by the RF and the row targets (Actual Not responders/Responders) are the actual values. Classification accuracy represents the proportion of the instances that were classified correctly (Actual Not responders and Predicted Not responder + Actual Responder and Predicted Responder/Total sample). NPI_f*s_, Neuropsychiatric Inventory frequencies and severity of symptoms.

## Discussion

We investigated the best predictors able to forecast the efficacy of rehabilitative intervention according to multimodal approach on cognitive and behavioral aspects in AD-continuum conditions. While recent evidence supports the effectiveness of these interventions (Baglio et al., 2015; Realdon et al., 2016; McDermott et al., 2019; Di Tella et al., 2020; Cafferata et al., 2021), little is known about the associated neural reserve mechanisms underlying cognitive and behavioral functions recovery. We identified brain reserve neuroimaging biomarkers and clinical features associated with the best rehabilitative outcomes, thus giving the opportunity to both clinicians and the healthcare system to exploit the available resources at best, by selecting the best tailored rehabilitative interventions for each patient. Despite the large consensus regarding the impact of cognitive and brain reserve in coping with age-related diseases (Valenzuela and Sachdev, 2006; Cabeza et al., 2018), a paucity of studies is available on the prognostic significance of structural brain measures in neurorehabilitation. To our knowledge, this is the first study focusing on the effects of brain reserves on the efficacy of rehabilitation interventions in AD-continuum conditions.

Considering the cognitive outcome of rehabilitation, our findings show that patients with low cognitive residual capabilities (MMSE level) at the time of admission and a high PB reserve in the parietal hemispheres (i.e., the normalized volume of AD-related parietal areas according to Ekman et al., 2018) are the best candidates to benefit from the rehabilitative treatment by achieving a significant improvement in global cognitive level. Neither FB nor MTL areas play a crucial role in the prediction of the cognitive outcome of intervention in our cohort of individuals.

The PB index (sum of volumes in: posterior cingulate, precuneus, superior parietal, inferior parietal, supramarginal gyrus) is not yet importantly compromised by the pathology progression as MTL index (Ekman et al., 2018) and can sustain the rehabilitation process in the mild to moderate stage of AD by integrating cognition, physical, and social activities (e.g., Venna et al., 2014). Such regions are importantly involved in the focusing of attention in internally directed cognition processes through the “tuning” of brain network activity and in the retrieval of autobiographical memories and in the planning of future acts (Zhang and Li, 2010; Leech and Sharp, 2014), somatosensory processing and visuo-spatial perception (Studer et al., 2014), socio-cognitive abilities (Rossetto et al., 2020; Tholen et al., 2020; Lion et al., 2021), and high-order processes (Culham and Kanwisher, 2001; Coull, 2004; Jubault et al., 2007; Desmurget and Sirigu, 2012). These aspects are well represented in the considered multimodal rehabilitation treatment, consisting in multifaced tasks touching cognitive, motor and social aspects of the patient’s wellbeing.

Interestingly, when exploring the contribution of lateralization, we observed that the right, but not left, PB areas alone are strongly associated with cognitive improvement after the multimodal rehabilitation. This confirms previous findings (Thompson et al., 2003; Karas et al., 2008; Derflinger et al., 2011; Cabinio et al., 2018; Yang et al., 2019) that report an asymmetrical degeneration of gray matter in AD, in terms of a greater atrophy of left than right hemispheres: the so called “left hemisphere susceptibility” (Shi et al., 2009; Donix et al., 2013). This is particularly true considering cortical thickness and surface areas in both amnestic mild cognitive impairment and mild AD. fMRI studies highlighted the role of bilateral activation as an effective way to counteract the effects of aging and neurodegeneration by reorganizing its function. In our previous work using fMRI we looked for a hypoactivation pattern in AD, and after a period of intensive multimodal rehabilitation, we found increased fMRI activation in some PB areas for restoring neural functioning (Baglio et al., 2015). We can assume that although there is some neural asymmetric deterioration occurring with the disease, the brain can increase bilateral neural activity to improve cognitive function recruiting residual areas from brain reserve.

Results herein also show that the best predictors to achieve a significant improvement in behavioral domain include the level of behavioral symptomatology at baseline, the volume of FB and PB areas, as well as sex. In details, lower brain volume in FB-index is associated with a greater probability to improve in the behavioral outcome. In fact, participants showing a high volume in FB-index are plausibly people without significant behavioral symptoms and are likely to remain stable over time. The behavioral symptoms associated with dementia are particularly disabling aspects of the disease, with a relevant impact on both patients and caregivers (Bessey and Walaszek, 2019). These symptoms include apathy, depression, anxiety, irritability, agitation, delusions, hallucinations, aberrant motor behavior, and appetite disorder. A recent study demonstrated that FB areas constitute the best predictor of behavioral impairment of dementia (Boublay et al., 2020), considering the strictly link between the frontal-limbic pathways in the etiopathogenesis of the main behavioral symptoms of the disease. In fact, changes in behavior represent a mark of the disease linked with impairment in executive functions and subserved by frontal lobe degenerative damage. Our findings indicate that the parietal reserve may also trigger a mechanism of improvement during rehabilitation even when a frontal degeneration is evident in behavioral aspects of AD-continuum. Accordingly, a paradigm shift is currently leading to new approaches in neurorehabilitation for older adults, favoring a functional-led multimodal method to enhance a wide range of cognitive functions, such as art-based tools. This approach is based on evidence supporting the potential benefit of the modulation of neural activity in brain areas that are better preserved in the aging process, such as parietal areas (Prakash et al., 2014). In particular, art-based stimulation resulted in a high effectiveness on different domains of functioning in AD, by acting on emotional channels and brain areas in which overactivation is observed in older adult age (Bucks and Radford, 2004; Klein-Koerkamp et al., 2012; Savazzi et al., 2020). Finally, a predictive role of sex on the behavioral outcome was found as females were more likely to benefit from rehabilitation. To the best of our knowledge no consistent data are available on sex and the prognostic rehabilitation effect in AD. Only pilot evidence suggests that sex may influence the cognitive effectiveness of motor treatment: older females show greater cognitive benefits from exercise than males (Barha and Liu-Ambrose, 2018). However, the mechanisms underlying this finding are still unknown.

This study is not without limitation: only one MRI examination per patient has been carried out at baseline. Subsequent studies may investigate neural plasticity induced by rehabilitation programs. Another limitation consists in the restricted neuropsychological battery considered for the outcome measures, not including measures with high ecological validity, which could have prevented the study from additional significant findings. Finally, our results should be interpreted with caution also considering that the effects of rehabilitation programs are variable depending on different factors, related to the contents of the program, the ability of therapists, the compliance of the participants. However, the relatively large sample size of the study renders the work relevant in the neurorehabilitation field. Future works will refine the predictive models by considering additional variables, such as the symptom duration and biomarkers (TAU protein and genotypes). Also, more sophisticated models including other proxy measures of cognitive reserve (employment/socio-behavioral indices) should investigate the possible mediating and/or moderating role of these variables explaining the association between treatment response and brain reserve.

These results indicate that cognitive behavioral improvement fostered by non-pharmacological treatments (Crescentini et al., 2014; Stinear et al., 2014), strictly depends on the actual brain reserve and functions of patients. This evidence supported the concept that structural characteristics of the brain have a protective role in AD. Cognitive and behavioral status alone are not sufficient to identify best responders to a multidomain rehabilitation treatment. Increased neural reserve, especially in the parietal areas, is relevant for the compensatory mechanisms activated by rehabilitative treatment. These data support clinical decision by identifying target patients with high probability of success after rehabilitative programs on cognitive and behavioral functioning.

## Conclusion

Our findings suggest that increased neural reserve, especially in the posterior brain structures, is a relevant predictor of the response to a rehabilitative treatment based on a holistic approach. Finally, baseline assessment of neural reserve indexes is fundamental to support clinical decision by identifying those patients that might most benefit from of multidomain rehabilitation.

## Data Availability Statement

The raw data supporting the conclusions of this article will be made available by the authors, without undue reservation.

## Ethics Statement

The studies involving human participants were reviewed and approved by the Don Gnocchi Ethics Committee. The patients/participants provided their written informed consent to participate in this study.

## Author Contributions

FB, MCl, MS, and SS conceived the work. FS, FR, and MA collected the data. MCa, SI, and SD performed the analyses. FB, SD, SI, MCa, and VB interpreted the results. FB, SD, SI, and MCa wrote the draft of the manuscript. All authors read and reviewed the final version of the manuscript.

## Conflict of Interest

The authors declare that the research was conducted in the absence of any commercial or financial relationships that could be construed as a potential conflict of interest.

## Publisher’s Note

All claims expressed in this article are solely those of the authors and do not necessarily represent those of their affiliated organizations, or those of the publisher, the editors and the reviewers. Any product that may be evaluated in this article, or claim that may be made by its manufacturer, is not guaranteed or endorsed by the publisher.

## Footnotes

1
http://surfer.nmr.mgh.harvard.edu/

2
http://enigma.ini.usc.edu/protocols/imaging-protocols
