# Supplementary material for: Neuroimaging Biomarkers Predicting the Efficacy of Multimodal Rehabilitative Intervention in the Alzheimer’s Dementia Continuum Pathology
Source: Front Aging Neurosci. 2021 Nov 22;13:735508. doi: 10.3389/fnagi.2021.735508 (PMC8645692; doi:10.3389/fnagi.2021.735508)
Supplement: Supplementary file 1 [file Table_1.docx]

**Neuroimaging Biomarkers predicting the efficacy of multimodal rehabilitative intervention in the AD continuum pathology**

**Supplementary Materials**

**Table S1:** Baseline characterisctics of treatment responders vs not responders at MMSE.

|  | ΔMMSE responder  (51%)  M | SD | ΔMMSE not responder  (49%)   M | SD | *p* | *effect size*  (*Cohen's d)* | *power* |
| --- | --- | --- | --- | --- | --- | --- | --- |
| Age (years) | 76.19 | 5.67 | 75.80 | 4.95 | 0.741 | 0.073 | 0.062 |
| Education (years) | 8.57 | 3.90 | 9.63 | 3.68 | 0.212 | 0.278 | 0.237 |
| Hamilton T0* | 5.70 | 4.08 | 7.55 | 4.86 | 0.099 | 0.412 | 0.378 |
| MMSE T0 (0-30) | 22.29 | 4.00 | 24.40 | 3.93 | 0.018 | 0.533 | 0.664 |
| FAS T0 | 22.12 | 10.25 | 24.13 | 10.89 | 0.393 | 0.190 | 0.136 |
| CAT T0 | 23.02 | 8.65 | 25.70 | 11.12 | 0.226 | 0.269 | 0.226 |
| NPI_f*s_ T0 (0-144) | 12.29 | 8.63 | 13.23 | 10.38 | 0.661 | 0.099 | 0.072 |
| NPI_distress_ T0 (0-60) | 6.51 | 4.01 | 6.84 | 5.63 | 0.764 | 0.068 | 0.060 |
| Estimated Total Intracranical Volume (mm^3^) | 1492000.00 | 163834.88 | 1525000.00 | 148808.20 | 0.355 | 0.205 | 0.151 |
| Z-MTB_global_ | -0.18 | 0.17 | -0.20 | 0.11 | 0.545 | 0.134 | 0.092 |
| Z-MTB_lh_ | -1.83 | 1.64 | -1.93 | 1.09 | 0.740 | 0.073 | 0.062 |
| Z-MTB_rh_ | -1.62 | 1.76 | -1.90 | 1.28 | 0.417 | 0.180 | 0.127 |
| Z-PB_global_ | -1.23 | 1.33 | -1.87 | 1.37 | 0.034 | 0.476 | 0.566 |
| Z-PB_lh_ | -1.12 | 1.30 | -1.59 | 1.26 | 0.103 | 0.365 | 0.372 |
| Z-PB_rh_ | -1.21 | 1.31 | -1.96 | 1.46 | 0.016 | 0.542 | 0.678 |
| Z-FB_global_ | -1.36 | 1.78 | -1.78 | 1.82 | 0.290 | 0.235 | 0.183 |
| Z-FB_lh_ | -1.50 | 1.85 | -1.90 | 1.90 | 0.337 | 0.213 | 0.159 |
| Z-FB_rh_ | -1.19 | 1.70 | -1.62 | 1.73 | 0.256 | 0.253 | 0.204 |
| Z-SBCB_global_ | -0.48 | 0.95 | -0.82 | 0.67 | 0.065 | 0.413 | 0.455 |
| Z-SBCB_lh_ | -0.52 | 1.01 | -0.88 | 0.67 | 0.064 | 0.415 | 0.458 |
| Z-SBCB_rh_ | -0.42 | 0.95 | -0.74 | 0.76 | 0.096 | 0.373 | 0.385 |

Hamilton T0 = Hamilton Depression Scale; MMSE T0 = Mini-Mental State Examination at baseline; FAS T0 = Phonological Fluency at baseline; CAT T0 = Categorial Fluency at baseline; NPI_f*s_ T0= Neuropsychiatric Inventory frequencies and severity of symptoms at baseline; NPI_distress_ T0= Neuropsychiatric Inventory caregiver distress; Z-MTB_global_ = Z-values of Medial Temporal Brain index; Z-MTB_lh_ = Z-values of left Medial Temporal Brain index; Z-MTB_rh_ = Z-values of right Medial Temporal Brain index; Z-PB_global_ = Z-values of Posterior Brain index; Z-PB_lh_ = Z-values of left Posterior Brain index; Z-PB_rh_ = Z-values of right Posterior Brain index; Z-FB_global_ = Z-values of Frontal Brain index; Z-FB_lh_ = Z-values of left Frontal Brain index; Z-FB_rh_ = Z-values of right Frontal Brain index; Z-SBCB_global_ = Z-values of Subcortical Brain index; Z-SBCB_lh_ = Z-values of left Subcortical Brain index; Z-SBCB_rh_ = Z-values of right Subcortical Brain index.

**Table S2:** Baseline characterisctics of treatment responders vs not responders at NPI.

|  | ΔNPI responder  (52%)  M | SD | ΔNPI not responder  (48%)   M | SD | *p* | *effect size*  (*Cohen's d)* | *power* |
| --- | --- | --- | --- | --- | --- | --- | --- |
| Age (years) | 76.86 | 5.57 | 75.30 | 4.78 | 0.186 | 0.299 | 0.261 |
| Education (years) | 9.21 | 3.85 | 9.00 | 3.90 | 0.810 | 0.054 | 0.057 |
| Hamilton T0* | 6.74 | 5.22 | 6.60 | 3.88 | 0.908 | 0.029 | 0.052 |
| MMSE T0 (0-30) | 23.77 | 4.23 | 22.57 | 3.86 | 0.191 | 0.296 | 0.256 |
| FAS T0 | 24.23 | 11.15 | 21.70 | 9.96 | 0.291 | 0.238 | 0.183 |
| CAT T0 | 25.12 | 10.05 | 22.73 | 9.61 | 0.283 | 0.242 | 0.187 |
| NPI_f*s_ T0 (0-144) | 15.37 | 9.33 | 9.70 | 8.82 | 0.007 | 0.623 | 0.784 |
| NPI_distress_ T0 (0-60) | 7.44 | 5.06 | 5.75 | 4.44 | 0.122 | 0.354 | 0.339 |
| Estimated Total Intracranical Volume (mm^3^) | 1487000.00 | 164910.42 | 1533000.00 | 141029.04 | 0.189 | 0.297 | 0.258 |
| Z-MTB_global_ | -0.17 | 0.13 | -0.22 | 0.14 | 0.154 | 0.322 | 0.295 |
| Z-MTB_lh_ | -1.74 | 1.26 | -2.14 | 1.39 | 0.182 | 0.302 | 0.265 |
| Z-MTB_rh_ | -1.60 | 1.41 | -2.06 | 1.56 | 0.173 | 0.308 | 0.274 |
| Z-PB_global_ | -1.38 | 1.35 | -1.86 | 1.25 | 0.105 | 0.368 | 0.367 |
| Z-PB_lh_ | -1.15 | 1.25 | -1.71 | 1.19 | 0.043 | 0.461 | 0.528 |
| Z-PB_rh_ | -1.48 | 1.41 | -1.82 | 1.32 | 0.266 | 0.251 | 0.198 |
| Z-FB_global_ | -1.50 | 1.88 | -1.76 | 1.65 | 0.511 | 0.148 | 0.100 |
| Z-FB_lh_ | -1.62 | 1.95 | -1.91 | 1.71 | 0.472 | 0.162 | 0.110 |
| Z-FB_rh_ | -1.35 | 1.79 | -1.57 | 1.60 | 0.561 | 0.131 | 0.089 |
| Z-SBCB_global_ | -0.53 | 0.76 | -0.80 | 0.88 | 0.138 | 0.336 | 0.316 |
| Z-SBCB_lh_ | -0.57 | 0.81 | -0.86 | 0.88 | 0.119 | 0.353 | 0.344 |
| Z-SBCB_rh_ | -0.48 | 0.80 | -0.72 | 0.91 | 0.207 | 0.285 | 0.241 |

Hamilton T0 = Hamilton Depression Scale; MMSE T0 = Mini-Mental State Examination at baseline; FAS T0 = Phonological Fluency at baseline; CAT T0 = Categorial Fluency at baseline; NPI_f*s_ T0= Neuropsychiatric Inventory frequencies and severity of symptoms at baseline; NPI_distress_ T0= Neuropsychiatric Inventory caregiver distress; Z-MTB_global_ = Z-values of Medial Temporal Brain index; Z-MTB_lh_ = Z-values of left Medial Temporal Brain index; Z-MTB_rh_ = Z-values of right Medial Temporal Brain index; Z-PB_global_ = Z-values of Posterior Brain index; Z-PB_lh_ = Z-values of left Posterior Brain index; Z-PB_rh_ = Z-values of right Posterior Brain index; Z-FB_global_ = Z-values of Frontal Brain index; Z-FB_lh_ = Z-values of left Frontal Brain index; Z-FB_rh_ = Z-values of right Frontal Brain index; Z-SBCB_global_ = Z-values of Subcortical Brain index; Z-SBCB_lh_ = Z-values of left Subcortical Brain index; Z-SBCB_rh_ = Z-values of right Subcortical Brain index.
